# 210 A project to promote the teaching of independent cycling to children with ambulant Cerebral Palsy in Ireland

**DOI:** 10.1093/eurpub/ckae114.127

**Published:** 2024-09-26

**Authors:** Aisling Renshaw

**Affiliations:** Enable Ireland, Ireland

## Abstract

The purpose of this project is to promote the teaching of cycling to children with ambulant Cerebral Palsy (CP). Research in Australia has shown that a lower proportion of children with ambulant CP can ride compared to typically developing peers and those that could, learn later.

For children with CP, there are numerous health benefits to independent cycling such as overall cardiovascular fitness and weight-management, sense of empowerment and achievement of a common childhood goal. It can be an additional mobility option and a great family activity option. It is possible that balance could improve and it may be beneficial to have exercise options which are low-impact and reduced weight-bearing. The mental health benefits also include improved sense of self-esteem and confidence alongside opportunities for social interaction and friendships.

The target group for this project are a wide range of stakeholders, from therapists working with children with CP; sports inclusion officers; Cycle Right trainers and families of children with ambulant CP.

Project aims to target a broad spectrum of ways for people to access support for learning to cycle.

During the development of this project, a small focus group was formed with parents and children with CP who had learnt independent cycling. Cycling can be an activity which fits into a family and child-centred approach to therapy. The benefits of their new skills was discussed under the themes of the ‘F words’ of disability. An initial teaching You-Tube video was made for children with milder co-ordination difficulties. A further video is planned teaching the more specific points when working with children with hemiplegia or asymmetrical diplegia. A workshop was developed for physiotherapists focusing on how to teach cycling skills to children with CP. Further links are being established with other stakeholders to maximise roll-out of the resources and workshops.

Cycling is such a fun and important activity and some specific skills are required when teaching, especially when the children have hemiplegia. This project aims to spread these teaching skills to a wide audience in the hope of as many children with CP in Ireland learning how to cycle as possible.

